# Effective Long-term Pediatric Pegvisomant Monotherapy to Final Height in X-linked Acrogigantism

**DOI:** 10.1210/jcemcr/luad028

**Published:** 2023-06-15

**Authors:** Christine P Burren, Georgina Williams, Edward Coxson, Márta Korbonits

**Affiliations:** Department of Paediatric Endocrinology and Diabetes, Bristol Royal Hospital for Children, University Hospitals Bristol and Weston NHS Foundation Trust, Bristol BS1 3NU, UK; Bristol Medical School, Department of Translational Health Sciences, University of Bristol, Bristol BS8 1UD, UK; Department of Paediatric Endocrinology, Noah's Ark Children's Hospital for Wales, Cardiff CF14 4XW, UK; Department Paediatrics, Royal United Hospital, Bath BA1 3NG, UK; Centre for Endocrinology, William Harvey Research Institute, Barts and the London School of Medicine, Queen Mary University of London, London EC1M 6BQ, UK

**Keywords:** X-linked acrogigantism, gigantism, growth hormone receptor antagonist, pegvisomant

## Abstract

X-linked acrogigantism (X-LAG) is characterized by extreme tall stature from early childhood resulting from duplication of the *GPR101* gene, in turn resulting in GH excess. Most cases present with pituitary tumors secreting GH and prolactin. Diffuse pituitary hyperplasia is uncommon and normal prolactin is rare. We present a girl with tall stature from 3 years of age; her height was +4.25 SD score at 5 years, with no signs of syndromic disease. She had significant GH excess, serum IGF-1 4 times the upper limit of normal and normal circulating GHRH, with normal pituitary magnetic resonance imaging over 13 years. No abnormalities were found in either the *AIP* or *MEN1* genes. Treatment with somatostatin analogues and dopamine agonists showed minimal therapeutic benefit, but significant side effects. She tested positive for duplication of *GPR101* 6 years after the initial diagnosis. She was then initiated on pegvisomant aged 12 years, achieving prompt IGF-1 normalization and growth cessation. Aged 16.5 years, she showed escape from IGF-1 control, and height velocity increased, but this responded well to a dose increase in pegvisomant, with reassuring long-term pediatric safety over 7 years. Her final height is +2.9 SD score. Currently, life-long pegvisomant treatment is planned with genetic counselling regarding future offspring.

## Introduction

Acromegaly in adults is a rare disorder. The even rarer parallel entity of GH excess during childhood is labeled pituitary gigantism or acrogigantism, in which open skeletal growth plates lead to tall stature as a central phenotypic component. Several genetic mechanisms have been identified for GH excess, especially in children or young people. Identified genetic causes of endocrine tumor syndromes (McCune-Albright syndrome and rarely MEN1, MEN4, and SDH-related syndromes) account for the minority, whereas familial isolated pituitary adenoma covers a greater proportion and includes 2 genes, *AIP* [1] and *GPR101* [2], although most familial isolated pituitary adenoma kindreds do not demonstrate a known genetic abnormality. Changes in *GPR101* as a cause of acrogigantism has only been reported in 36 cases to date [3-5]. Most patients with X-linked acrogigantism (X-LAG) have a microduplication at Xq26.3, involving the *GPR101* gene, coding for an orphan G protein–coupled receptor most likely involved in the regulation of the GH axis. The underlying genetic mechanism has recently been described: the duplication disrupts the normal chromatin architecture in this area, allowing previously unavailable regulatory regions to influence the *GRP101* promoter, creating a new topologically associating domain (TAD) around the *GPR101* gene [6]. This has established X-LAG as the first endocrine “TADopathy”, and explains the significant (more than 100×) upregulation of gene expression observed in these patients. The most notable clinical clue for the diagnosis of X-LAG is age of onset of excess linear growth before the age of 5 years, most often from infancy, with 1 patient even identified in utero with a pituitary tumor [7]. All patients carrying the *GPR101* duplication appear to develop acrogigantism.

Early diagnosis and successful sustained control of GH excess are both crucial for optimal short- and long-term outcomes. In childhood, where excess linear growth cannot be reversed, and in adulthood, to reduce the consequences of uncontrolled acromegaly, such as joint problems, structural damage to bones, hypertension, cardiovascular disease, type 2 diabetes, sleep apnea, psychological problems, and increased mortality.

The management of isolated GH excess in childhood is complex. Because the majority of patients have large pituitary adenomas, surgery is the initial therapeutic focus, whereas adjuvant medical therapy and, in some cases, radiotherapy are needed in a minority. Pharmacological options, either preoperatively or following surgery, target GH secretion and tumor growth (somatostatin analogues [SSA], dopamine agonists) or block GH action (GH-receptor antagonists). We describe a girl with *GPR101* duplication and marked GH and IGF-1 excess but with no pituitary mass. GH suppression was not achieved by somatostatin and dopamine agonists, whereas switching to GH receptor blockade achieved prompt biochemical and clinical control. Pegvisomant monotherapy over the long duration in childhood was safe and effectively limited final height.

## Case Presentation

A female patient had tall stature from age 3 years and presented to endocrine services aged 5 years. Several secondary teeth had erupted aged 4 years, and greasy skin with acne was reported from 4.5 years. She was otherwise well. There was a medical history of maternal preeclampsia with emergency cesarean section at 32 weeks. Her birth weight was 1.59 kg (−0.25 SD score [SDS]). There was no family history of tall stature or endocrine neoplasia. Physical examination at presentation showed a height of 129.3 cm (height SDS +4.25, target height SDS +1.3), weight 27 kg (+2.68 SDS), head circumference 57 cm (+3.0 SDS), mildly coarse facial features, and blocked pores on forehead skin. She was prepubertal with no breast development, no axillary hair but stage 2 pubic hair (abnormal for age).

## Diagnostic Assessment

**Figure 1.**
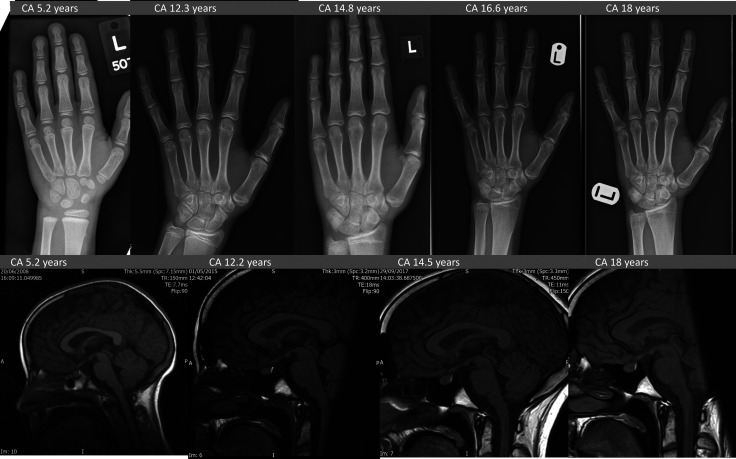
Serial pituitary MRI and bone age radiology. Upper panel shows serial bone age X-rays. (A) At diagnosis chronological age (CA) 5.2 years, bone age (BA) 6.9 years. (B) At the start of pegvisomant treatment CA 12.3 years, BA 13.5 years. (C) Stable on pegvisomant, BA advancement resolved CA 14.8 years, BA 15 years. (D) Linear growth had resumed CA 16.6 years, BA 16 years. (E) At final height CA 18 years, BA 17.6 years (ie, mature). Lower panel: 4 of 10 serial pituitary MRI scans. All demonstrate slightly bulky but not enlarged pituitary of homogenous texture and enhancement; appearance and size and unchanged throughout the 13-year period. Sagittal T1-weighted images with gadolinium contrast (F) at diagnosis, (G) at the start of pegvisomant treatment at CA 12.3 years, (H) 2.3 years after pegvisomant treatment started, (J) 5.8 years after pegvisomant treatment started; (K) at CA 18 years.

Biochemical investigations at presentation are detailed in Table 1. She showed a significantly elevated basal GH levels with lack of suppression on oral glucose tolerance test, serum IGF-1 was 4 times age-adjusted upper limit of normal. Her circulating GHRH level was normal. The LH releasing hormone stimulation test showed prepubertal response. The rest of the pituitary function was normal. An advanced bone age of 6.9 years was documented at a chronological age of 5.2 years (Fig. 1). Serial thin-sliced contrast enhanced pituitary magnetic resonance imaging (MRI) scans over 13 years showed an unchanged marginally bulky pituitary, but no mass lesion (Fig. 1). Genetic testing found normal female karyotype 46XX, and no *AIP, MEN1*, or *GNAS* gene variants were identified in her blood-derived DNA. See further genetic assessment results in the following section during the follow-up period.

**Table 1.** Biochemical results at presentation

Test	Result			Normal range for appropriate age
TSH	2.2 mIU/L			0.3-4.0
Free T4	19.2 pmol/L (1.49 ng/dL)			10-24
Prolactin	493 mIU/L (23.2 μg/L)			<700 (32.9)
IGF-1	79 nmol/L (604 μg/L)			4-20 (30.6-153)
GH baseline	38 µg/L			0-5
OGTT (1.75 g/kg glucose) Time, min GH, µg/L Glucose, mmol/L (mg/dL)	Time 0' 30’ 60’ 90’ 120’ 180’	GH 38 18 16 26 22 20	Glucose 5.4 (97) 7.5 (135) 6.1 (110) 4.9 (88) 6.6 (119) 4.4 (79)	Peak glucose <11 (198) Nadir GH <0.4
GHRH	<60 ng/L			< 60 ng/L
ACTH	22.5 pmol/L (102 pg/mL)			7-51 (31.8-232)
Cortisol	192 nmol/L (6.95 µg/dL)			100-400 (3.6-14.5)
LHRH test Time (min) LH (IU/L) FSH (IU/L)	Time 0’ 20’ 60’	LH<0.5 2.0 1.2	FSH<0.5 1.4 2.5	Test performed at age 5.25 y These results show prepubertal response
Urinary steroid profile	Mild degree of adrenarche			

Abbreviation: LHRH, LH releasing hormone; OGTT, oral glucose tolerance test.

## Treatment

**Figure 2.**
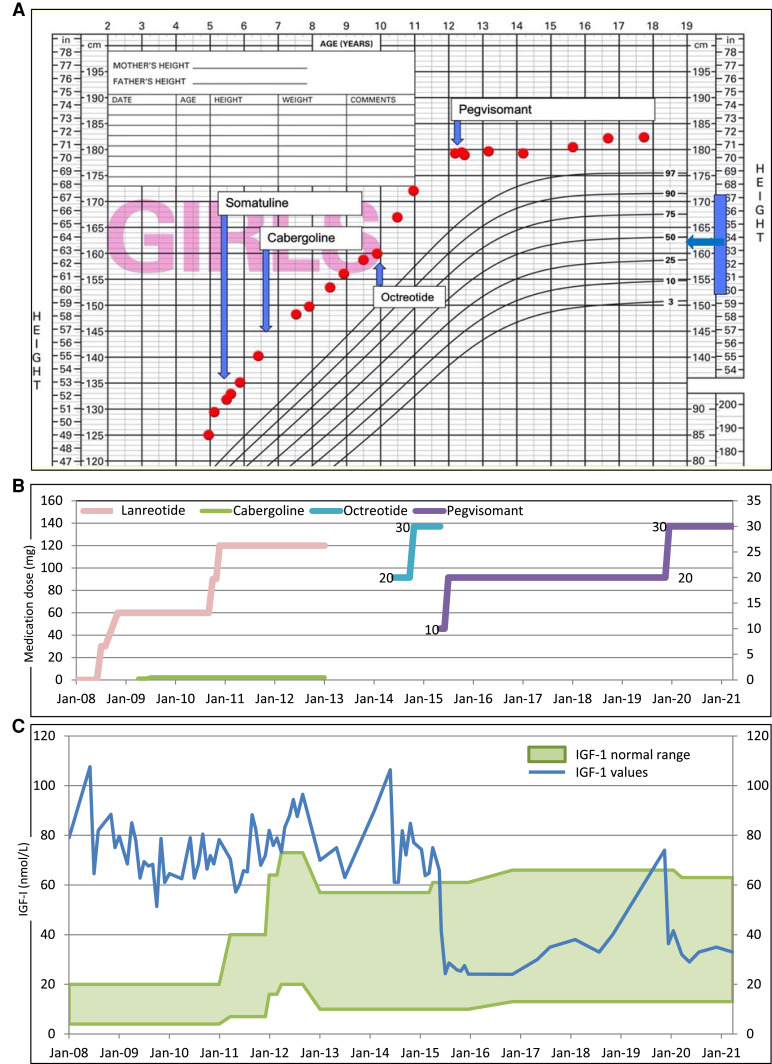
(A, upper panel) Growth chart, (B, middle panel) timeline of medications and (C, lower panel) serial serum IGF1. (A) Growth chart shows height (cm) plotted from aged 5 to 18 years, horizontal arrow on right hand side of panel A indicates mid-parental height accompanied by vertical blue bar to indicate target centile range; vertical arrows indicate start date of each of the 4 medications (lanreotide [Somatuline], cabergoline, octreotide LAR, and pegvisomant), with horizontal label width visually indicating duration of each therapy. (B) Plots the 4 medications showing respective dose increases: lanreotide doses plotted against left hand y-axis (dose increments 30, 60, 90, and 120 mg/mo), whereas remaining 3 medication doses plotted against right hand y-axis: cabergoline dose increments 0.25 and 0.5 mg/wk (lower green line), octreotide (blue line) dose increments 20 mg then 30 mg/mo for 12 months. Pegvisomant (purple line) commenced at 10 mg/d, rapidly escalated to 20 mg then increased to 30 mg ongoing. (C) Serum IGF-1 plotted against y-axis (nmol/L, conversion factor to µg/L is 7.65) over 13 years (x-axis). Blue line indicates serial values of the patient and green shaded band encompasses normal age ranges. IGF-1 normal range differs according to age. In addition, serum IGF-1 was measured at 4 different laboratories over the 13 years, which accounts for the undue fluctuation in the green band of normal range. Panels B and C have same x-axis time scale to illustrate IGF-1 response to respective medications (ie, persistently elevated IGF-1 throughout somatostatin analogue and cabergoline treatments) and then prompt IGF-1 reduction on introduction of pegvisomant followed by brief escape during puberty, when dose increase to 30 mg pegvisomant per day rapidly regained control.

Treatment commenced aged 5.3 years with monthly injections of first generation SSA lanreotide Autogel, applied in increasing doses from 30 to 120 mg. This resulted in minimal IGF-1 reduction and no height velocity reduction (11.1 cm/y, Fig. 2). After 10 months, cabergoline (250 mcg twice weekly) was added aged 6.1 years. The following 6-month interval height velocity (5.1 cm/y) was briefly encouraging, but not sustained. Clinically meaningful growth slowing did not occur, despite lanreotide and cabergoline dose escalations (Fig. 2) over 4.6 years; gastrointestinal symptoms (abdominal discomfort, flatulence) persisted. Treatment ceased aged 9.9 years because of minimal efficacy yet ongoing side effects, and her abdominal symptoms resolved. For 6 months, height velocity remained acceptable (4.4 cm/y).

However, her height velocity then increased aged 11 years (10.8 cm/y), partially attributable to puberty. She developed daily headaches, nausea, backache, and hand cramps. Repeat oral glucose tolerance test aged 11.1 years confirmed ongoing GH excess (baseline 16.2 µg/L, nadir 8.9 µg/L). A different long-acting SSA (octreotide LAR, 30 mg) was commenced because of the lack of other available treatment options at the time. In response to therapy, her serum IGF-1 initially reduced from 106.4 nmol/L (811 µg/L) to 61.1 nmol/L (467; normal range for ages 9.8-57.2 years [75-437.5]), suggesting biochemical responsiveness, but 12 months of treatment did not normalize IGF-1 nor slow growth.

## Outcome and Follow-up

By age 11 years, 3 medical therapies had been unsuccessful, and her predicted final height was >193 cm. Fortuitously, ongoing research revealed chromosome Xq26.3 microduplication, encompassing *GPR101*, as a cause of childhood-onset GH excess [2], and droplet-digital PCR and high-density array comparative genomic hybridization confirmed this genetic abnormality, as a de novo change, in our case [8]. This X-LAG diagnosis confirmed an intrinsic underlying genetic mechanism, providing compelling evidence for funding approval for pegvisomant, a GH-receptor blocker.

At aged 12.2 years (bone age 13.5 years, European shoe size 44.5), pegvisomant was started (10 mg titrated to 20 mg once daily by subcutaneous injection) and was well tolerated and effective. Biochemical improvement was prompt (Fig. 2B): serum IGF-1 became normal by 2 weeks and was in the lower half of the normal range by 6 weeks 24.3 nmol/L (186 µG/L; 10.7-60.7 [81.8-464]). Clinical improvement occurred as headache, nausea, back pain, and hand discomfort resolved. There was a decrease in soft-tissue enlargement, quantified by a reduction in ring size from size P at the time when pegvisomant was started to ring size N, representing 2-unit drop in the ring size chart. Height did not appreciably increase during the first 3 years of pegvisomant treatment.

At review aged 16.5 years, her documented height was 182.1 cm, a 2-cm increase over 12 months, at a relatively mature bone age and despite excellent compliance with pegvisomant 20 mg daily. She started to take an oral contraceptive pill for contraceptive purposes at the age of 16.2 years. Biochemistry revealed an elevated IGF-1 74 nmol/L (566 µg/L; 20-63 [153-482]), and this pegvisomant increased to 30 mg: IGF-1 promptly normalized and no further height increase occurred. At aged 18 years, her final height 182.1 cm (+2.9 SDS), with mature bone age and she is well, without side effects including normal liver function. Daily injections of pegvisomant monotherapy continue, and we envisage life-long pegvisomant monotherapy to prevent GH excess. In due course, genetic counseling regarding offspring will be offered.

## Discussion

This case describes a now 19-year-old female with X-LAG, with extreme tall stature emerging aged 3 years, and a germline *GPR101* mutation identified aged 11 years [8], on pegvisomant from age 12 years. X-LAG is extremely rare, with only 36 cases currently reported [3-5]. This report adds longer term follow-up data on unusual X-LAG features and adolescent phase pegvisomant through to final height. Absence of an adenoma in X-LAG is uncommon (20%) [3], and serial MRI imaging over 13 years provides reassurance of a stable entity. Only 4, including this case, of the 36 known cases of X-LAG where data are available show no hyperprolactinemia [3, 5]. Nevertheless, intrinsic GH excess was problematic with extreme tall stature and nonresponsiveness to multiple medical treatments, highlighting challenges in medically managing X-LAG.

The GH receptor antagonist pegvisomant is well-established in acromegaly management. The ACROSTUDY provides predominantly adult long-term safety efficacy data on 2090 patients [9]: mean treatment duration (7.6 years) is similar to our case (6 years). Within ACROSTUDY, 0.7% (n = 15) started pegvisomant <18 years, although treatment duration and genetic diagnosis were not reported [9]. Pediatric reports involving pegvisomant in X-LAG patients show encouraging response out to 3 to 5 years’ treatment duration [3, 10].

Pegvisomant is excellent in controlling IGF-1, whereas it has no effect on tumor control. A solely medical focus without preceding surgery is uncommon in X-LAG (10% had no adenoma, but most if these still had surgery) and in pituitary-origin GH excess in general (ACROSTUDY only 24% had no initial surgery). Previously, before the availability of effective medical treatments, an X-LAG case like ours with hyperplasia was treated with hypophysectomy [8]. Because our patient had normal pituitary functions apart from the GH axis, surgery was not a preferred therapeutic option. Our patient's lack of GH response to first-generation SSA and cabergoline resonates with other pegvisomant-treated X-LAG cases [8]. Pegvisomant was usually added to existing therapies in combination, whereas our patient ceased SSA 1 year earlier because of side effects and lack of efficacy, so uniquely demonstrates the response to initiating pegvisomant as monotherapy in childhood.

This case illustrates pegvisomant's efficacy in X-LAG to limit final height outcome. We recommend considering commencing pegvisomant earlier in pediatric GH excess. Within this positive message is the cautionary note that appreciable linear growth in X-LAG can occur at relatively mature bone maturation, indicating the importance of tight biochemical control. Effective pegvisomant use is generally defined as IGF-1 normalization. Our case's IGF-1 escaped to 1.2× upper limit of normal at a late stage of linear growth at the age of 17 years. Our data would support tighter targets (IGF-1 in the lower half of normal range) in pegvisomant therapy during the growth years for patients with X-LAG. Current pegvisomant dose is 30 mg and it is unclear if our case's GH production will remain the same or somewhat decline with age, as observed in patients with acromegaly in general [9].

## Learning Points

- Pegvisomant monotherapy is effective as monotherapy in adolescent years of X-linked acrogigantism (X-LAG).
- X-LAG usually includes a pituitary mass (80%) and elevated prolactin (89%), but even in the absence of these, control of the intrinsic GH excess can be medically challenging.
- Linear growth response to X-LAG driven GH excess can be significant even at relatively mature skeletal ages with incompletely fused epiphyses.
- Pegvisomant dose optimization should target IGF-1 to the lower half of normal range in adolescents, even at late stages of bone maturation to best control linear growth and requires close monitoring.

## Abbreviations

MRI: magnetic resonance imaging
SDS: SD score
SSA: somatostatin analogue
X-LAG: X-linked acrogigantism
